# Seq-InSite: sequence supersedes structure for protein interaction site prediction

**DOI:** 10.1093/bioinformatics/btad738

**Published:** 2024-01-11

**Authors:** SeyedMohsen Hosseini, G Brian Golding, Lucian Ilie

**Affiliations:** Department of Computer Science, University of Western Ontario, London, ON N6A 5B7, Canada; Department of Biology, McMaster University, Hamilton, ON L8S 4K1, Canada; Department of Computer Science, University of Western Ontario, London, ON N6A 5B7, Canada

## Abstract

**Motivation:**

Proteins accomplish cellular functions by interacting with each other, which makes the prediction of interaction sites a fundamental problem. As experimental methods are expensive and time consuming, computational prediction of the interaction sites has been studied extensively. Structure-based programs are the most accurate, while the sequence-based ones are much more widely applicable, as the sequences available outnumber the structures by two orders of magnitude. Ideally, we would like a tool that has the quality of the former and the applicability of the latter.

**Results:**

We provide here the first solution that achieves these two goals. Our new sequence-based program, Seq-InSite, greatly surpasses the performance of sequence-based models, matching the quality of state-of-the-art structure-based predictors, thus effectively superseding the need for models requiring structure. The predictive power of Seq-InSite is illustrated using an analysis of evolutionary conservation for four protein sequences.

**Availability and implementation:**

Seq-InSite is freely available as a web server at http://seq-insite.csd.uwo.ca/ and as free source code, including trained models and all datasets used for training and testing, at https://github.com/lucian-ilie/Seq-InSite.

## 1 Introduction

Proteins are among the most influential molecules in a cell, being responsible for many functions, such as DNA replication, gene expression, protein synthesis, intercellular communication, etc. (Jones and Thornton 1996). Proteins interact with each other for proper function; therefore, it is crucial to study them in the context of their interacting partners. These interactions involve the binding of two or more proteins to form a complex structure and detecting these can contribute to discovering many protein functions (Jansen *et al.* 2003).

In order to fully comprehend the molecular mechanisms of protein–protein interactions, it is essential to identify the specific residues within proteins that facilitate the formation of interactions. Protein interactions can be detected experimentally or predicted by computational methods. Although experimental methods such as immunoprecipitation (Lin and Lai 2017), pull-down assay (Louche *et al.* 2017), surface plasmon resonance (Douzi 2017), bacterial two-hybrid (Karimova *et al.* 2017) and cytology two-hybrid (Atmakuri 2017) may generate a more accurate description of interaction sites, they are expensive and time consuming. Computational methods are fast and cheap and can provide useful information to be used as such or to generate the most likely candidates to be confirmed experimentally. Improving the quality of computational predictions is thus a fundamental problem (Zhang and Kurgan 2018, Casadio *et al.* 2022, Soleymani *et al.* 2022).

Protein interaction site prediction is the problem of predicting the locations of a given protein where interactions are likely to take place. It involves using bioinformatics algorithms and machine learning techniques to analyse large amounts of protein interaction data. Various machine learning techniques have been used for this problem, such as logistic regression, random forest, support vector machines and neural networks, etc. (Zhang and Kurgan 2018, Casadio *et al.* 2022). In spite of considerable progress, much work is needed to achieve the necessary accuracy that can have a great impact on the understanding of the molecular mechanisms.

Computational models can be classified into two large categories, according to the type of information used as input: sequence or structure. Structured-based methods use the 3D structures of proteins to predict interaction sites (Zeng *et al.* 2020, Wang *et al.* 2022, Yuan *et al.* 2022). The main drawback of these methods is the limited availability of protein structures. On the other hand, sequence-based methods (Zhang and Kurgan 2019, Li *et al.* 2021, Hosseini and Ilie 2022, Manfredi *et al.* 2023) rely solely on the protein sequences, thus having the advantage of being applicable to a far broader range of proteins, as the number of available protein sequences outnumbers that of structures by two orders of magnitude (Qiu *et al.* 2020). The majority of models utilize supervised feature extractions in order to create some form of representation for each amino acid including position-specific scoring matrix (PSSM), hidden Markov models (HMM) and a dictionary of protein’s secondary structure (DSSP).

The rise of embeddings in natural language processing, from contextual independent word2vec (Mikolov *et al.* 2013) or GloVe (Pennington *et al.* 2014) to the more effective context dependent models such as ELMo (Peters *et al.* 2018) and Bert (Devlin *et al.* 2018) enabled the effective extraction of information via self-supervised learning from enormous amounts of unlabelled data in the readily utilizable form of numerical vector embeddings associated with input words. This had implications in the field of proteomics as well (Iuchi *et al.* 2021, Ofer *et al.* 2021), where large amounts of unlabelled sequences were already available (UniProt 2019). Protein embeddings associate vectors in high-dimensional space with residues and many models have been proposed, including ProtVec (Asgari and Mofrad 2015), SeqVec (Heinzinger *et al.* 2019), PRoBERTa (Nambiar *et al.* 2020), MSA-transformer (Rao *et al.* 2021), ESM-1b, ESM2 (Rives *et al.* 2021), ProtBert, ProtXLNet, ProtAlbert, ProtT5 (Elnaggar *et al.* 2021). These protein embeddings have many important applications in various areas, e.g. structure prediction (Senior *et al.* 2020, Yang *et al.* 2020, Jumper *et al.* 2021) or function prediction (Kulmanov and Hoehndorf 2020, Gligorijević *et al.* 2021, Lai and Xu 2022).

Our contribution in this paper is three-part. First, we introduce our new model, Seq-InSite (Sequence-based Interaction Site prediction), that uses the ProtT5 and the MSA-transformer embeddings in an ensemble architecture model that combines a multi-layer perceptron (MLP) and an LSTM. The result is a model that surpasses the current state-of-the-art sequence-based programs by a wide margin and performs on par with or better than the current state-of-the-art structure-based models. Second, we provide thorough testing and comparison with many models on both newest and most widely used datasets. This not only assesses the performance of Seq-InSite, but also offers a comprehensive perspective on the current state of the area. Third, we present applications where the predictive capabilities of Seq-InSite are used and evaluated in the context of evolutionary conservation of four important proteins.

Our new model comes as a continuation of a series of improvements. We first used a ProtVec-derived embedding in DELPHI (Li *et al.* 2021), together with a CNN-RNN ensemble architecture and many input features. In PITHIA (Hosseini and Ilie 2022), we used the MSA-transformer embeddings with an attention-based architecture, and showed that the embeddings alone successfully replace the multitude of features previously used by all programs. Manfredi *et al.* (2023) noticed with ISPRED-SEQ that different embeddings, such as ProtT5 and ESM1-b (Rives *et al.* 2021), produce better results, surpassing PITHIA’s. With Seq-InSite, we reached the level where it is possible to obtain structure-level quality predictions using only the widely available protein sequences.

## 2 Materials and methods

### 2.1 Datasets

We use a large selection of datasets, including three benchmark datasets that have been used frequently in the field, Dset_186, Dset_72 (Murakami and Mizuguchi 2010), and Dset_164 (Dhole *et al.* 2014) and three most recent datasets, Dset_500 (Hosseini and Ilie 2022), Dset_448 (Zhang and Kurgan 2018), and its subset, Dset_336, that consists of protein-binding proteins.

As the number of available protein structures is limited, structure-based methods face the challenge of finding a good quality dataset for training procedure. This limitation resulted in using the 422 proteins in datasets Dset_186, Dset_164 and Dset_72 to both train and test. For instance, DeepPPISP, EGRET and HN-PPISP use a test set consisting of 70 out of the 422 available proteins and the 352 remaining protein as training. Similarly, GrapPPIS and RGN make use of a test set of 60 proteins, training on the rest; we are using these datasets as well as Dset_60 and Dset_70.

GraphPPIS introduced also Test_315, a dataset created based on the newly solved protein complexes in PDB (January 2014–May 2021) by removing any overlapping or similar protein structures with any proteins in Dset_186, Dset_72, or Dset_64; for uniformity, we shall call Test_315 as Dset_315.

#### 2.1.1 Training and validation datasets

The training dataset was produced as follows. We took all 22 654 proteins from the most recent version of PiSite (Higurashi *et al.* 2009) (January 2019) and removed all sequences with no interaction residues or containing <50 amino acids; 14 203 sequences remained after this elimination. We then used PSI-CD-HIT (Fu *et al.* 2012) to remove any sequences that have any similarity above 25%, as customary in this area, with any of the testing datasets, Dset_72, Dset_164, Dset_186, Dset_448 and Dset_500. The remaining proteins, 11 523, form Seq-InSite’s training dataset; this is further split into training (99%) and validation (1%) sets; this size of validation data is sufficient given that our dataset has about 2.9 million samples.

Table 1 gives an overview of all these datasets.

**Table 1.** Datasets overview; all but the last one are testing datasets.

Dataset	Proteins sequences	Mean length	Total residues	Interacting	
				Residues	%
Dset_500	500	274.54	137 270	21 222	15.50
Dset_448	448	260.05	116 500	15 810	13.60
Dset_336	336	252.80	84 941	15 810	18.60
Dset_315	315	207.40	65 331	9355	14.32
Dset_186	186	194.72	36 219	5517	15.23
Dset_164	164	197.00	32 308	5749	17.79
Dset_72	72	213.90	15 401	1768	11.48
Dset_70	70	168.44	11 791	2332	19.78
Dset_60	60	219.06	13 144	2075	15.78
Train+validate	11 523	251.90	2 902 667	545 724	18.80

### 2.2 Input embeddings

The transformer architecture has been successful in producing superior protein folding models, including the highly appraised AlphaFold (Jumper *et al.* 2021), which uses multiple sequence alignment in a supervised manner. MSA-transformer (Rao *et al.* 2021) also uses attention and multiple sequence alignment to generate powerful 768D amino acid embeddings in an unsupervised manner. The T5 language model (Raffel *et al.* 2020), a variant of the transformer architecture, has demonstrated its effectiveness in large-scale natural language processing. ProtT5-XL (Elnaggar *et al.* 2021) uses a T5 architecture with the Bert masking procedure to compute amino acid embeddings. It trains a T5 model on the BFD dataset (Steinegger and Söding 2018, Steinegger *et al.* 2019) and then fine tunes it on Uniref50 (Suzek *et al.* 2015). Elnaggar *et al.* (2021) also developed a larger model, ProtT5-XXL, but increasing the size did not improve its performance. Our tests showed that ProtT5-XL (hereby called ProtT5) is the superior model for predicting interaction sites. Its embeddings have size 1024.

ProtT5 is particularly useful for proteins with long sequences or those that do not have a high-quality multiple sequence alignments, thanks to its gigantic and state-of-the-art architecture that can represent any amino acid with a high-quality embedding. On the other hand, MSA-transformers can capture more complex relationships between protein sequences that have a high-quality alignments by utilizing the existing relations among the aligned protein sequences.

Our new program, Seq-InSite, uses both MSA-transformer and ProtT5 embeddings as inputs to better capture the complexity of protein sequences and achieve improved performance. The architecture of Seq-InSite is described in detail in the next section.

### 2.3 Model architecture

**Figure 1.**
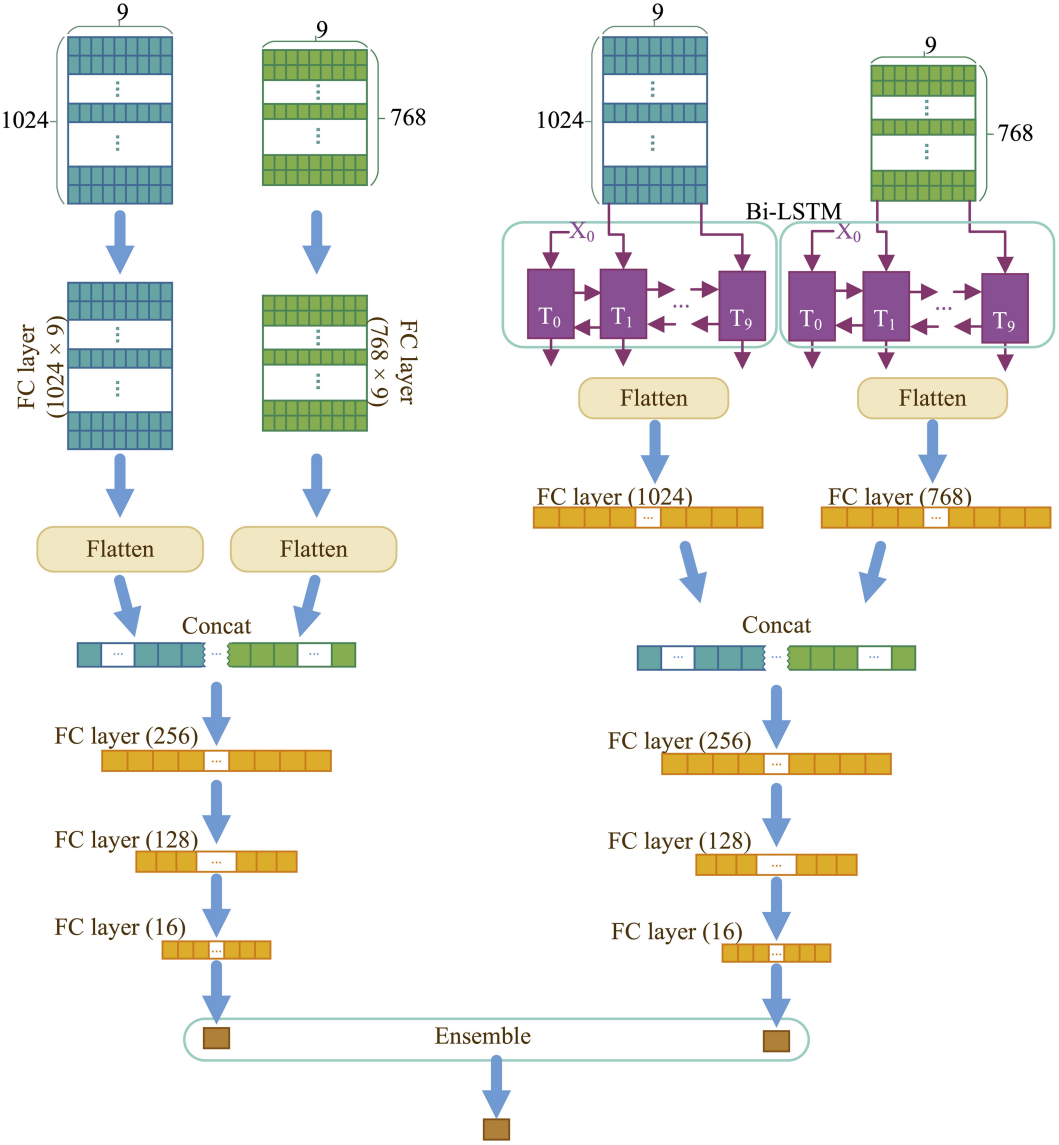
The architecture of Seq-InSite.

Seq-InSite’s architecture is inspired by ensemble learning, with two MLP and LSTM components that represent an ensemble of both ProtT5 and MSA-transformer embeddings. These components are fused together through multiple dense layers to predict protein interaction sites. The architecture is depicted in Fig. 1 and the parameters are given in Table 2.

**Table 2.** Parameters of the model.

Model	Parameters	Values
MLP	Layer sizes	1024×9 , 768×9, 256, 128, 16, 1
RNN	LSTM unites	64
	Fully connected unit	64, 1
	Activation function	Tanh
All	Batch size	1024
	Dropout rate	0.3
	Optimizer	Adam $\left(\beta_{1}=.9;\beta_{2}=.999\right)$
	Loss function	Binary cross entropy
	Learning rate	0.001
	Patience (early stop)	4

Seq-InSite uses a many-to-one structure, where a window centred on each target amino acid is used to gather information from neighbouring residues in an attempt to predict interaction propensity. The window size, w=9, has been determined experimentally. The sequence’s ends are padded with zeros.

#### 2.3.1 Architecture of MLP network

The MLP component, shown in the left side of Fig. 1, contains a fully connected layer, a flatten layer and four fully connected layers with dropout. The input size is w×e, where *w* is the window size and *e* is the embedding size that depends on the language model being used (768 for MSA-transformer, 1024 for ProtT5), and uses the sigmoid activation function in the final layer to generate output probabilities, while all other layers use ReLU activation function.

#### 2.3.2 Architecture of RNN network

The RNN component of Seq-InSite, depicted on the right side of Fig. 1, uses a bidirectional LSTM layer with 64 units, followed by a flatten layer and two fully connected layers that reduce the dimensionality to one, with input size varying depending on the language model being used, and the LSTM layer set to return a sequence of values.

#### 2.3.3 Architecture of ensemble network

The final model uses an ensemble network to combine information from both the MLP and LSTM components to predict the interaction propensity of each amino acid in a protein sequence. The MLP and LSTM branches process the input data separately, using MSA-transformer and ProtT5, and produce their own predictions. The final prediction is obtained by averaging the predictions from the two branches to improve accuracy and reliability. The architecture of the model is shown in Fig. 1.

### 2.4 Implementation

The code is implemented using Keras (keras.io) with TensorFlow GPU (Abadi *et al.* 2016) as the backend. It utilizes generators to handle the large data, for which reason local shuffling is used, since generators read small parts of the fasta file at a time. A batch size of 1024 is used to enable multiple proteins to be included in each batch. By using generators, we significantly reduced the memory requirement. The program requires 110 GB of RAM, and the training process takes about 25 min per epoch. During testing, the program takes about 18 s to process a sequence if embeddings are already available. Computing MSA-transformer embeddings takes 10–20 min; ProtT5 embeddings require approximately 40 s.

### 2.5 Model selection

Multiple models, involving combinations of MLP, RNN (LSTM), CNN and Transformer architectures, were constructed and evaluated using the training dataset. No testing data was used to choose the final model. Each model was trained on 99% of the available data, and its performance was evaluated using the remaining 1% validation data; see Table 1. The final Seq-InSite model was selected based on its performance, as measured by the area under the PR curve, on the validation data. We wanted a metric that is threshold independent, to indicate overall performance, and, of the two such candidates, the area under the ROC curve and the area under the PR curve, the latter represents a better metric for the performance of an algorithm when data is skewed (Davis and Goadrich 2006, He and Garcia 2009, Saito and Rehmsmeier 2015). We would like to add that the area under the ROC curve selects the same model as well, which makes the model choice even more robust.

## 3 Results

### 3.1 Competing methods

Despite relying solely on sequence data to predict interaction sites, we compared our model not only to sequence-based methods but also to state-of-the-art structure-based methods. We selected for comparison a number of methods based on the quality of their performance as well as suitability for our study regarding code availability, reproducibility of their results and testing datasets used. More details are given in the Supplementary Material. The methods considered include sequence-based models: CRFPPI (Wei *et al.* 2015), DELPHI (Li *et al.* 2021), DLPred (Zhang *et al.* 2019), D-PPIsite (Hu *et al.* 2023), ISPRED-SEQ (Manfredi *et al.* 2023), LORIS (Dhole *et al.* 2014), PIPENN (Stringer *et al.* 2022), PITHIA (Hosseini and Ilie 2022), PSIVER (Murakami and Mizuguchi 2010), SCRIBER (Zhang and Kurgan 2019), SPPIDER (Porollo and Meller 2007), SPRINGS (Singh *et al.* 2014), SPRINT (Taherzadeh *et al.* 2016) and SSWRF (Wei *et al.* 2016); and structure-based models: AttentionCNN (Lu *et al.* 2021), DeepPPISP (Zeng *et al.* 2020), EGRET (Mahbub and Bayzid 2022), GraphPPIS (Yuan *et al.* 2022), HN-PPISP (Kang *et al.* 2023), MaSIF (Gainza *et al.* 2020), ProB-site (Khan *et al.* 2022) and RGN (Wang *et al.* 2022).

We encountered many problems while attempting to run the code of some of the methods above, which prevented us from performing full comparison on all datasets. We could not run AttentionCNN, EGRET and HN-PPISP, and our requests to the corresponding authors were not answered. For RGN, the (pretrained) code provided in the website performed not as good as claimed in their paper. The authors responded to our request and provided a different pretrained model, which we ran and reported its results. Running the available code of D-PPIsite and ProB-site produced very different results from those published. The authors responded to our messages, but the discrepancies could not be corrected.

### 3.2 Evaluation metrics

As good practice in binary classification evaluation, we use many metrics: sensitivity (or recall), precision, specificity, accuracy, F1-score, Matthew’s correlation coefficient (MCC), area under the receiver operating characteristic (ROC) curve and area under the precision–recall (PR) curve. The area under the PR curve is best suited to represent the performance of an algorithm when data is skewed (Davis and Goadrich 2006, He and Garcia 2009, Saito and Rehmsmeier 2015). According to common practice used in many previous studies, the threshold was set such that the number of predicted interaction residues equals the number of actual interaction residues, resulting in the same value for sensitivity, precision and F1-score.

### 3.3 Performance comparison

**Figure 2.**
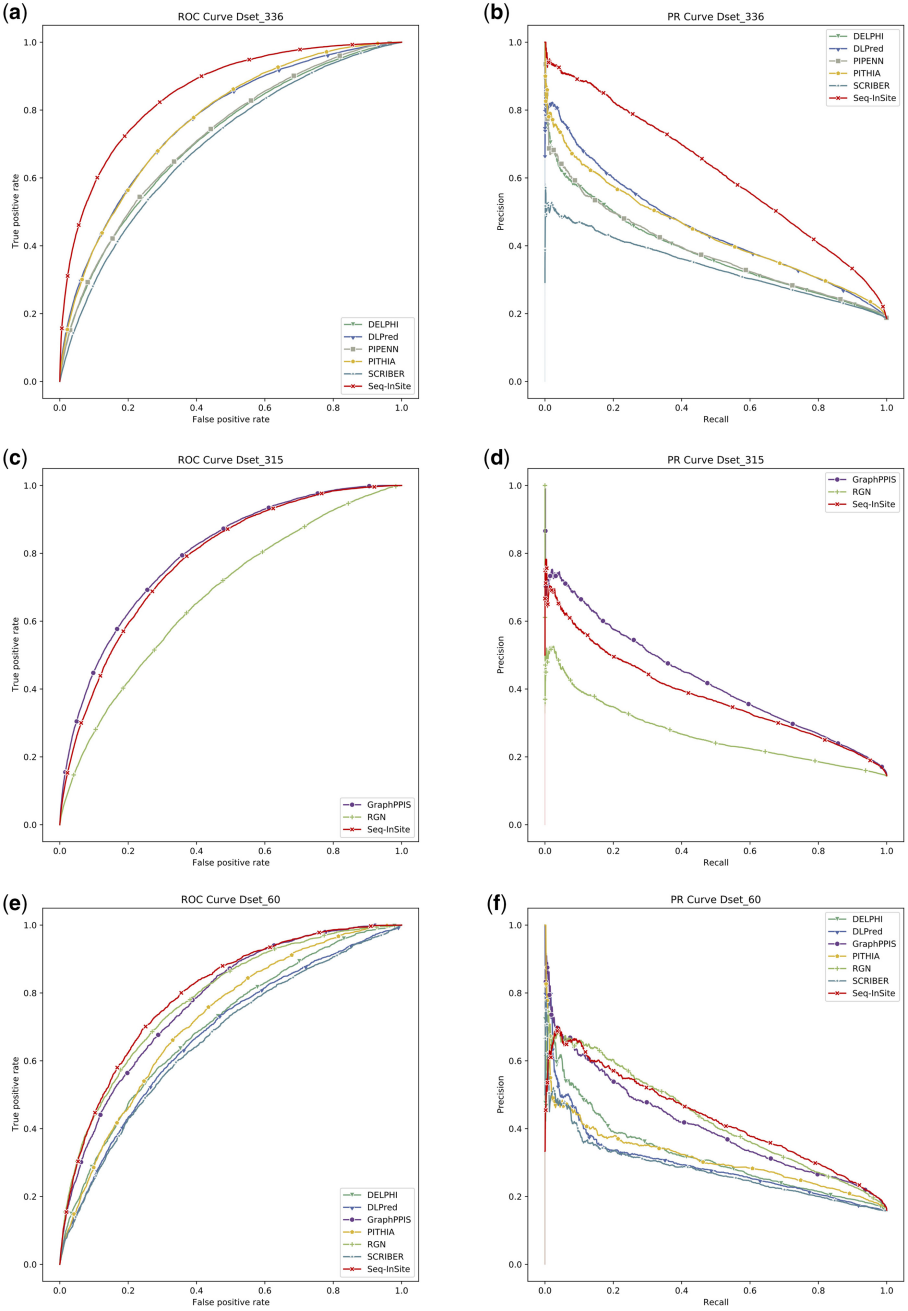
ROC and PR curves for the tests sets (a) and (b) Dset_336, (c) and (d) Dset_315, and (e) and (f) Dset_60.

Comparisons are shown in Table 3 as one heatmap per dataset. The ROC and PR curves are given in Fig. 2 for the models that could be run.

**Table 3.** Performance comparison.^a^

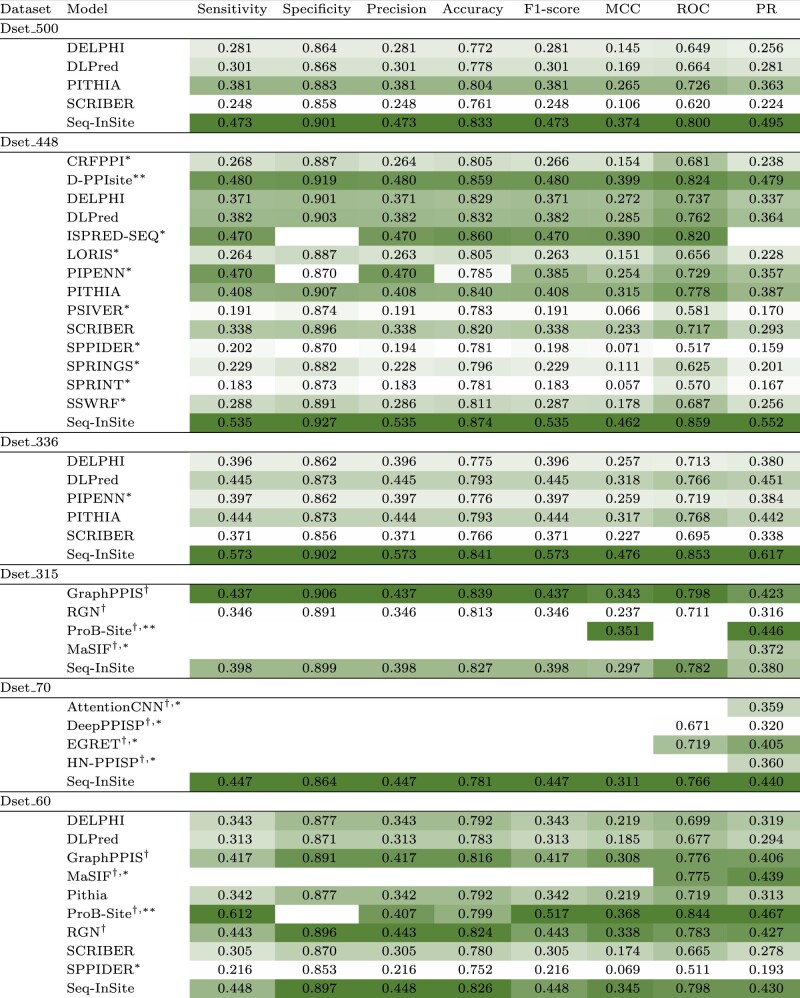

a The methods marked by

† are structure-based; the rest are sequence-based. The results for the methods marked by

* were taken from their papers; the results marked by

** could not be reproduced; the rest were computed by us; see text for details. Darker colour indicates better performance.

#### 3.3.1 Comparison against sequence-based models

Seq-InSite performs far better than all programs, with the exception of ISPRED-SEQ, which is much closer than the rest. Unfortunately, ISPRED-SEQ is available only as web server, with one sequence allowed per run. Therefore, we could not compute its PR area or include its curves. The PR plot for Dset_336 in Fig. 2 is particularly convincing for the very large advantage of Seq-InSite. Recall that Dset_336 is especially relevant as it contains protein-binding proteins; the remaining proteins in Dset_448 bind only ligands that are not proteins (Zhang and Kurgan 2018). For similar behaviour on Dset_500 and Dset_448, see Supplementary Table S1 and Supplementary Fig. S1b and d.

#### 3.3.2 Comparison against structure-based models

Due to the limitations explained earlier, we need to find a way to properly train our model for testing on datasets Dset_60, Dset_70 and Dset_315, such that we include as many protein sequences as possible in training, without having any similarity (above 25%) with testing data. Therefore, we had to adjust our trainign dataset for each of the three testing datasets. In that case, we started with the 14 203 proteins computed earlier (see Training and validation datasets) and removed all protein sequences that have more than 25% similarity with any sequence in a given testing dataset. The obtained training datasets are shown in Table 4. Each set is split into 99%/1% training and validation.

**Table 4.** Datasets used for training in order to test on Dset_60, Dset_70 and Dset_315.

Dataset	Proteins sequences	Mean length	Total residues	Interacting	
				Residues	%
Train+Valid. 60	14 035	233	3 270 769	640 694	19.58
Train+Valid. 70	14 031	233	3 274 194	639 958	19.54
Train+Valid. 315	13 690	232	3 181 794	625 718	19.66

The comparison on Dset_60 is very interesting because we managed to include both sequence-based and structure-based methods. The most striking feature, evident from the curve grouping in Fig. 2f, is the difference between sequence-based models and structure-based models, with the latter having a clear advantage. Our new method, Seq-InSite, is the exception, clearly belonging to the higher group, as the top performing method. Seq-InSite has the highest area under both the PR curve and the ROC curve.

The test on Dset_315 is the only test where Seq-InSite comes in second place, behind GraphPPIS; see Fig. 2c–d. Interestingly, when restricting the comparison to long proteins (length over 400), which are the most difficult to predict, Seq-InSite outperforms GraphPPIS; see Supplementary Table S2.

The MaSIF program is a more general tool that has been included for comparison by some papers (Yuan *et al.* 2022). However, its training set has large similarities with both Dset_60 (35 proteins, over half) and Dset_315 (104 proteins, one third). In spite of these large similarities, its performance is comparable with that of Seq-InSite.

We used Dset_70 to include some comparison with several programs we could not run. All we could do is compare the computed area under the PR and ROC curves of Seq-InSite with those reported in the corresponding papers. We were unable to test GraphPPIS on all proteins in Dset_70 because GraphPPIS did not compute the feature set for all proteins in the combined dataset of Dset_72, Dset_164 and Dset_186. Seq-InSite significantly outperforms the other methods, in spite of the fact that these are structure-based models.

### 3.4 Ablation study

The ablation study we performed in order to understand the importance of various components and inputs of Seq-InSite is presented in the Supplementary Table S3.

## 4 Evolutionary conservation

As an application of Seq-InSite’s predictive power, we analysed four different proteins. These are the alpha-subunit of the haemoglobin protein, the bacterial phosphoenolpyruvate carboxylase (PPC), the PPC’s four homologues in plants and the human BRCA1 protein.

For the haemoglobin proteins, we used the same dataset as in DELPHI (Li *et al.* 2021) to be able to compare the two results. The human haemoglobin was used for the query sequence (142 amino acids in length). For the other proteins, BLASTP was used to search for homologues. In the case of the bacterial PPC, the *Escherichia coli* PPC protein sequence was used as the query (883 amino acids in length). In the case of the plant PPCs, the four *Arabidopsis thaliana* duplicate sequences were used as the query (967, 963, 968, 1032 amino acids in length). There are many isoforms of the human BRCA1, and so the canonical form (UniProtKB database entry P38398; 1863 amino acids in length) was used as the query. The search was restricted to the RefSeq protein database to ensure good quality hits. The top 100 hits were taken but excluded hits from *Escherichia* and *Shigella* in the case of PPC and excluded hits from the genus Homo in the case of BRCA1. For each set, an additional cluster BLASTP was done (Steinegger and Söding 2017). This database clusters together sequences within 90% identity and 90% length to other members of the cluster. This analysis of clustered sequences provides a taxonomically broader range of hits. Again, the top 100 hits were taken. The query sequence, some selected homologues and the two sets of sequences from the top 100 hits were combined. Sequences were aligned using MUSCLE (Edgar 2004). Sequences that were identical, that contained unusually long stretches of inserted or deleted residues, or that were too distantly related to the query were manually trimmed with the aid of phylogenies constructed with IQ-TREE (Nguyen *et al.* 2015). The alignment lengths for the proteins were: haemoglobin protein alignment length 308, bacterial phosphoenolpyruvate carboxylase alignment length 980, the PPC’s four homologues in plants alignment length 1282, 1254, 1029, 1341, and the BRCA1 protein alignment length 2157.

**Figure 3.**
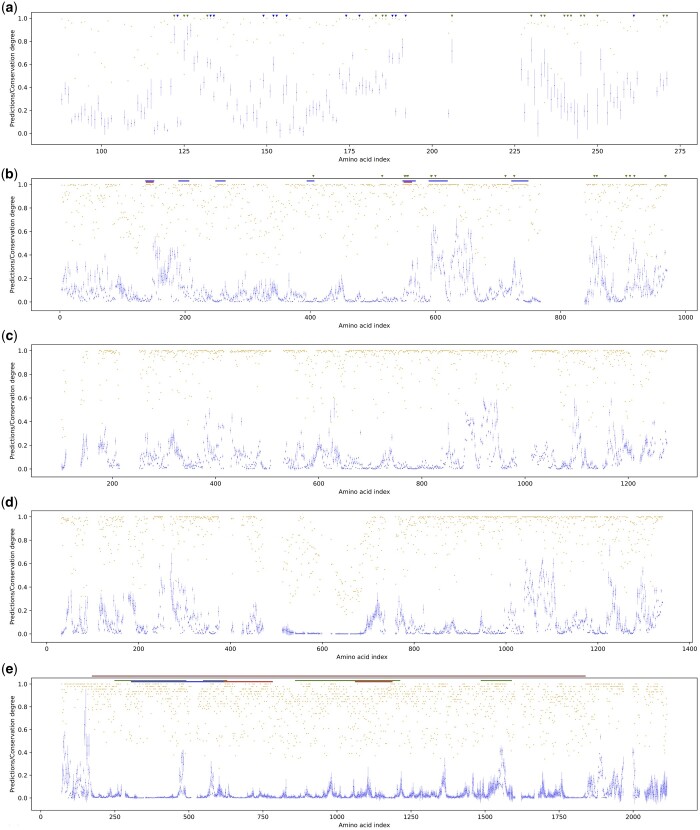
Interaction site predicted by Seq-InSite versus the degree in conservation. Seq-InSite predictions are shown in blue (mean in dark blue and variance in light blue) and degree of conservation in orange. The proteins are: (a) alpha haemoglobin—interfaces in green, binding sites in blue, (b) bacterial phosphoenolpyruvate carboxylase (PPC)—active sites in red, pepcarbxylase domains in blue and ligand binding sites in green, (c) and (d) PPC’s plant homologous and (e) human BRCA1 protein—interaction colours: intrinsic disorder in brown, DNA binding in red, p53 binding in blue, protein binding in green.

The results of Seq-InSite for the haemoglobin sequences are shown in Fig. 3a. The locations of important interfaces for the polypeptide interfaces to form a tetramer (db_xref=“CDD:271278”) are shown with coloured arrows.

It can be easily observed that the predicted PPI sites are in reasonable agreement with the actual sites involved in protein-to-protein interaction or protein sites binding to chemical moieties. The agreement is not perfect but primarily due to false negatives rather than false positives.

The bacterial PPC has a known 3D structure. The protein has two active sites, seven pepcarbxlase domains and 17 ligand binding sites, highlighted in Fig. 3b with coloured bars and arrows. The degree of agreement between these important sites and interaction probability within the plot can be observed. At the N-terminus there are none of these sites despite potentially high interaction probability which could be due to intra-peptide bindings.

Three of the plant PPCs (duplicates 1, 2 and 3) are very similar to the bacterial form. While there is no known 3D structure for plant PPCs, given their similarity it is doubtful that the structure would differ by very much and indeed, the predicted AlphaFold structure is similar. Since a BLASTP search using PPC1 query returned hits to PPC2 and PPC3, only the results for one (PPC1) is shown in Fig. 3c. The results for PPC4 are somewhat different and shown in Fig. 3d. A dotplot of PPC1 versus PPC4 shows a large insertion in PPC4 of approximately 100aa between amino acids 320 and 440 (466 and 718 in alignment) and shows poor similarity between the two proteins for the first 150 amino acids (235 aa in alignment). A PPI plot for PPC4 is shown in Fig. 3d and suggests that, from this data, the large insertion is unlikely to be involved in protein interactions.

The human BRCA1 protein (UniProtKB P38398) is a difficult test for Seq-InSite. This protein is 83% in an intrinsically disordered state. As a result, AlphaFold can only make poorly supported predictions for the majority of the length of the protein. Seq-InSite, on the other hand, makes strong predictions at the amino terminus of the protein and less strongly supported predictions scattered throughout the length of the protein (Fig. 3e). Like many other proteins with intrinsically disordered regions, the BRCA1 protein binds very promiscuously to a variety of other proteins. A few of its interactions are indicated in Fig. 3e with coloured bars.

## 5 Discussion and conclusion

We have introduced a new program for protein interaction site prediction, Seq-InSite. In spite of the fact that Seq-InSite is sequence-based, it succeeded in matching or outperforming the, usually, much more accurate structure-based programs. In all our tests, Seq-InSite came in the first place, with a single exception, when it placed second. Given the fact that sequences are much more readily available than structures, Seq-InSite can effectively replace structure-based methods for interaction site prediction.

Despite this accuracy, the predicted sites, as exemplified in Fig. 3, cannot always be validated from the biological sequences alone. This does not mean that these predictions are wrong but rather that much still might be learnt from the sequences.

It is interesting to note that the improved structure prediction from protein sequences, such as done by AlphaFold, may not bring the expected increase in use of structure in predicting various protein properties. This is due to the fact that the same mechanisms that enable the successful extraction and use of information from sequences to improve structure prediction can also help improve the prediction of other properties as well, directly from the sequence. Any information extracted from the sequence about structure and used subsequently as structure-based prediction, should be, at least in principle, used directly, bypassing structure prediction.

## Supplementary Material

btad738_Supplementary_Data

## Data Availability

In order to make Seq-InSite widely available, we have built a web server at http://seq-insite.csd.uwo.ca/, where the user provides protein sequences and receives the results by e-mail or directly in the browser. The source code, including running instructions, pre-trained models and all datasets used for training and testing, are available at https://github.com/lucian-ilie/Seq-InSite.
