# Supplementary material for: Seq-InSite: sequence supersedes structure for protein interaction site prediction
Source: Bioinformatics. 2024 Jan 11;40(1):btad738. doi: 10.1093/bioinformatics/btad738 (PMC10796176; doi:10.1093/bioinformatics/btad738)
Supplement: btad738_Supplementary_Data [file btad738_supplementary_data.zip › seqinsite_supp.pdf]

Seq-InSite: sequence supersedes structure for protein interaction site prediction

– Supplementary material –

SeyedMohsen Hosseini, G. Brian Golding, Lucian Ilie*

1 More comparisons

Dataset Dset_355 was created by Li *et al.* [1] as a subset of Dset_448 containing 355 proteins that have no significant similarity with the training set of DLPred. The performance of many programs on Dset_355 is shown in Supplementary Table 1. All programs, except DLPred, perform similarly on Dset_448 and Dset_355, as can be seen by comparing the results for Dset_448 in Table 3 of the manuscript with those for Dset_355 in Supplementary Table 1. Since we have all the data for Dset_355, we can plot the ROC and PR curves for Dset_355, and they are also indicative for the behaviour on Dset_448. They are shown in Supplementary Figure 1. We notice the very large advantage Seq-InSite has over the rest, especially in the PR plot on the right.

Supplementary Table 1: Comparison on Dset_355. ROC and PR curves are shown in Figure 1.

Model	Sens	Spec	Prec	Acc	F1	MCC	ROC	PR
CRFPPI*	0.245	0.898	0.245	0.820	0.245	0.143	0.662	0.214
DELPHI	0.368	0.914	0.368	0.849	0.368	0.282	0.749	0.333
DLPred	0.305	0.906	0.305	0.834	0.305	0.211	0.725	0.268
LORIS*	0.240	0.897	0.240	0.818	0.240	0.137	0.637	0.203
PITHIA	0.384	0.916	0.384	0.853	0.384	0.301	0.769	0.350
PSIVER*	0.177	0.888	0.177	0.803	0.177	0.065	0.583	0.155
SCRIBER	0.322	0.908	0.322	0.838	0.322	0.230	0.719	0.275
SPRINGS*	0.209	0.893	0.209	0.811	0.209	0.102	0.608	0.178
SPRINT*	0.167	0.887	0.167	0.801	0.167	0.054	0.571	0.150
SSWRF*	0.267	0.901	0.267	0.825	0.267	0.168	0.667	0.228
Seq-InSite	0.525	0.935	0.525	0.886	0.525	0.460	0.860	0.533

2 Comparison on Dset_315 by protein length

Dataset Dset_315 is the only test where Seq-InSite comes second best, behind the structure-based model GraphPPIS. We have analyzed in more detail the behaviour and noticed that Seq-InSite manages to outperform GraphPPIS for longer proteins, which are the more difficult case. The results are shown in Supplementary Table 2 where the data was divided by protein length in comparable bins of size 100.

*Corresponding author: `ilie@uwo.ca`

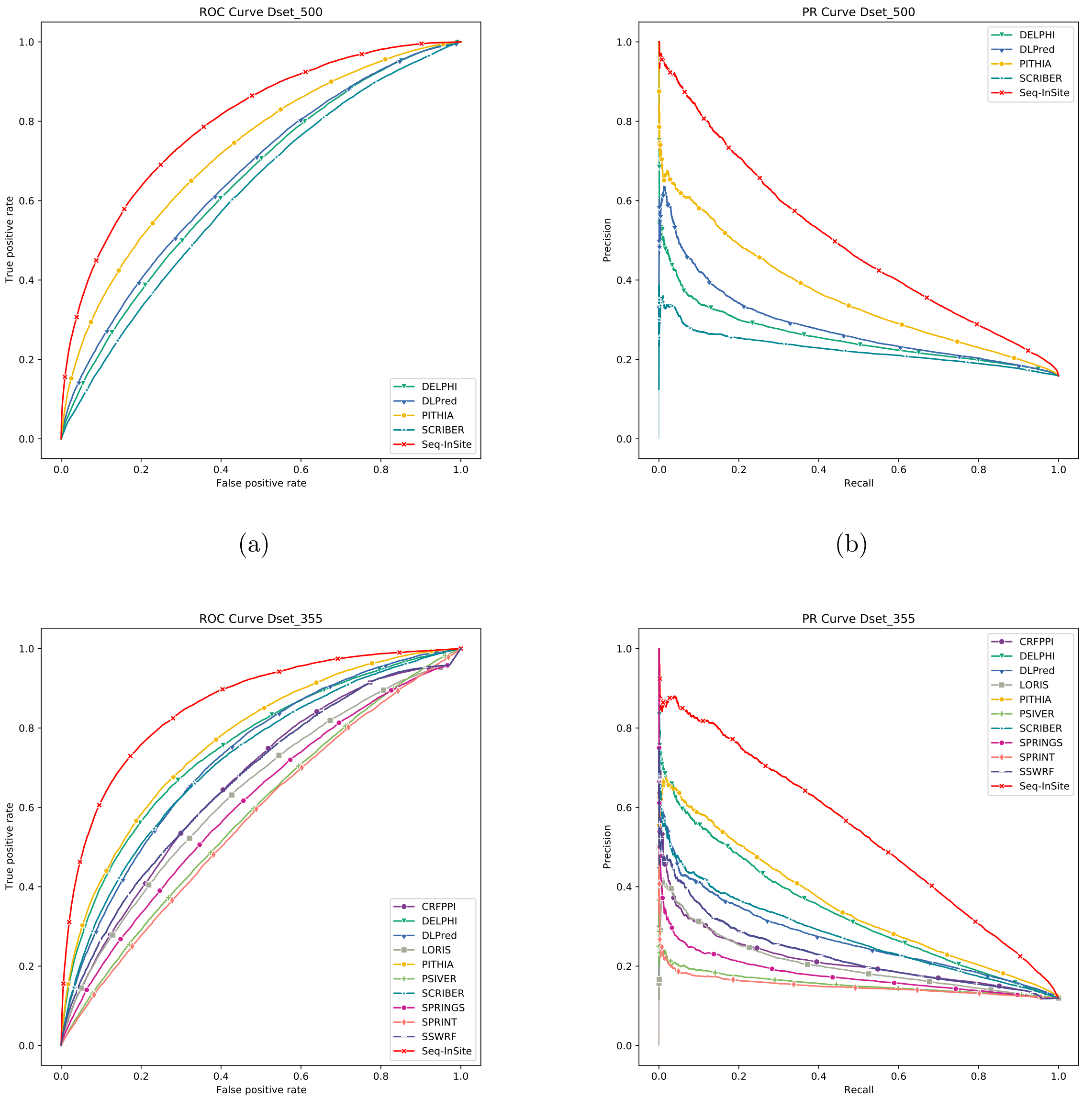

Supplementary Figure 1: ROC and PR curves for the results in Supplementary Table 1 for (a)-(b) Dset_500 and (c)-(d) Dset_355.

Supplementary Table 2: Comparison by length on Dset_315.

Length interval	Model	Sens	Spec	Prec	Acc	F1	MCC	ROC	PR	Proteins
0 - 100										64
	GraphPPIS	0.602	0.797	0.602	0.731	0.602	0.399	0.777	0.625	
	RGN	0.512	0.751	0.512	0.670	0.512	0.263	0.690	0.527	
	Seq-InSite	0.531	0.761	0.531	0.683	0.531	0.292	0.718	0.550	
100 - 200										127
	GraphPPIS	0.473	0.872	0.473	0.794	0.473	0.345	0.775	0.478	
	RGN	0.316	0.834	0.316	0.733	0.316	0.150	0.625	0.293	
	Seq-InSite	0.407	0.856	0.407	0.768	0.407	0.263	0.749	0.419	
200 - 300										62
	GraphPPIS	0.360	0.908	0.360	0.838	0.360	0.267	0.750	0.316	
	RGN	0.269	0.894	0.269	0.816	0.269	0.164	0.678	0.234	
	Seq-InSite	0.326	0.903	0.326	0.830	0.326	0.228	0.738	0.291	
300 - 400										31
	GraphPPIS	0.367	0.928	0.367	0.871	0.367	0.296	0.811	0.337	
	RGN	0.274	0.918	0.274	0.852	0.274	0.192	0.714	0.237	
	Seq-InSite	0.330	0.924	0.330	0.864	0.330	0.254	0.779	0.305	
400 -										27
	GraphPPIS	0.226	0.944	0.226	0.896	0.226	0.171	0.769	0.184	
	RGN	0.197	0.942	0.197	0.892	0.197	0.139	0.688	0.145	
	Seq-InSite	0.280	0.948	0.280	0.903	0.280	0.228	0.757	0.211	

3 Ablation study

We performed experiments aimed at clarifying the importance of various components and inputs of Seq-InSite. Combining the two architectural branches, MLP and LSTM, with the two input embeddings, MSA-transformer and ProtT5_XL (denoted MSA and T5 in these experiments), in all meaningful ways results in seven models, including the final ensemble. The results of testing all sub-models on Dset_448 are shown in Supplementary Table 3, where the names of the models are self explanatory. We conclude from the results that the ProtT5_XL embeddings bring more information than the MSA-transformer ones. With fixed architecture, ProtT5_XL produces better performance. For the two architectures, MLP and LSTM, the comparison is less clear. LSTM is better when a single embedding is used, but MLP takes the lead when both embeddings are used. Combining all four components in the final ensemble produces the best results, the Seq-InSite model.

Supplementary Table 3: Ablation study using Dset_448.

Model	Sens	Spec	Prec	Acc	F1	MCC	ROC	PR
MLP_MSA	0.379	0.903	0.379	0.832	0.379	0.282	0.761	0.362
MLP_T5	0.512	0.923	0.512	0.867	0.512	0.435	0.846	0.522
LSTM_MSA	0.422	0.909	0.422	0.843	0.422	0.331	0.780	0.403
LSTM_T5	0.517	0.924	0.517	0.869	0.517	0.441	0.847	0.523
MLP_MSA_T5	0.528	0.926	0.528	0.872	0.528	0.453	0.853	0.540
LSTM_MSA_T5	0.513	0.924	0.513	0.868	0.513	0.436	0.851	0.529
ENSEMBLE	0.535	0.927	0.535	0.874	0.535	0.462	0.859	0.552

References

[1] Y. Li, G. B. Golding, and L. Ilie. DELPHI: accurate deep ensemble model for protein interaction sites prediction. Bioinformatics, 37(7):896–904, 2021.
