# Supplementary material for: Seq-InSite: sequence supersedes structure for protein interaction site prediction
Source: Bioinformatics. 2024 Jan 11;40(1):btad738. doi: 10.1093/bioinformatics/btad738 (PMC10796176; doi:10.1093/bioinformatics/btad738)
Supplement: btad738_Supplementary_Data [file btad738_supplementary_data.zip › seqinsite_supp2_competing_methods.pdf]

Seq-InSite: sequence supersedes structure for protein interaction site prediction

– Supplementary material 2: Selection of competing methods –

SeyedMohsen Hosseini, G. Brian Golding, Lucian Ilie*

Protein interaction site prediction encompasses various subcategories, including protein-peptide interaction site prediction, protein-DNA interaction site prediction, compound-protein interaction site prediction. Our primary focus is on protein-protein interaction site prediction.

We conducted a Google Scholar search using the keywords "protein", "interaction site", and "prediction" and filtered the results to include papers published after 2017. We then reviewed the first twelve pages of search results and constructed Table 1. Each column in the table represents a feature that we regarded as essential for a paper to be considered for inclusion into our comparison tests.

Table 1: Methods considered: method name and reference, publication year, number of citations, datasets used in the paper, source code availability and reproducibility of results, web server availability.

**Method/ref.**	**Year**	**Cit.**	**Datasets**	**Code**	**Web server**
PPISP_MCDMD [23]	2017	9	CJ and BW	no	no
svm [8]	2018	41	Chen [4]	no	no
S4VM [33]	2019	10	Dset_170 [1]	no	no
DLPred [39]	2019	72	Dset(186, 164, 72)	yes/reprod.	not anymore
SCRIBER [40]	2019	93	Dset(186, 164, 72, 448)	no/reprod.	yes
DeepPPISP [38]	2020	177	Dset(186, 164, 72)	yes/reprod.	not anymore
SVM [28]	2020	2	generated(not known)	no	no
ConvsPPIS [41]	2020	37	DBv5 [29]	no	no
XGBoost [5]	2020	48	Dset_170 [1]	no	no
IntPred [20]	2020	70	generated	no	not anymore
CNN [36]	2020	39	DBv5 [29]	yes/not checked	no
AttentionCNN [16]	2021	5	Dset(186, 164, 72)	yes/not reprod.	no
randomForest [22]	2021	4	CJ [3] and BW [2]	no	no
PPISP-XGBoost [32]	2021	34	Dset(186, 164, 72)	yes/not reprod.	no
DeepPPISP-XGB [30]	2021	7	Dset(186, 164, 72)	yes/not reprod.	no
HANPPIS [27]	2021	5	Dset(186, 164, 72)	no	no
GraphPPIS [37]	2022	54	Dset(186, 164, 72, 315)	yes/reprod.	yes
RGN [31]	2022	4	Dset(186, 164, 72, 315)	yes/(semi)reprod.	no
EGRET [17]	2022	18	Dset(186, 164, 72)	yes/not reprod.	no
DGC_PRSA [15]	2022	0	Dset(186, 164, 72)	no	no
ctP2ISP [13]	2022	2	Dset(186, 164, 72)	yes/not reprod.	no
Prob-Site [12]	2022	3	Dset(186, 164, 72, 315)	yes/not reprod.	yes
HN-PPISP [11]	2023	8	Dset(186, 164, 72)	partly/not reprod.	no

*Corresponding author: ilie@uwo.ca

During our background research, we observed that a significant number of algorithms report their results using for testing the datasets Dset_186, Dset_164, Dset_72, and Dset_448. Consequently, we chose to concentrate on models that utilize these specific datasets for testing. These datasets are primarily employed in the realm of sequence-based interaction site prediction.

From the various methods that were applied to the Dset (186, 164, 72), we have chosen the ones that we were able to reproduce their results with our machines. Additionally, we examined the chosen papers and incorporated some of the models that were compared in those papers: CRFPPI [35], LORIS [6], PSIVER [19], SPRINGS [24], SPPIDER [21], SPRINT [26], SSWRF [34], MaSIF [7]. Furthermore, we explored papers that have cited the selected papers and took them into consideration as well: DELPHI [14], ISPRED-SEQ [18], PITHIA [9], PIPENN [25], D-PPIsite [10].

With the aim of highlighting the substantial disparity between sequence-based models and structure-based models, we sought out structure-based models that either employ these datasets or a subset for testing purposes. During our search, we discovered several methods that fulfilled our requirements, including DeepPPISP, GraphPPIS, RGN, EGRET, and more. These models proved to be suitable for our experimentation with sequence-based datasets, allowing us to conduct meaningful comparisons and assessments. Moreover, we found that GraphPPIS introduced a novel dataset labeled as Dset_315, which was predominantly designed for use by structure-based models such as RGN, EGRET, HN-PPISP, AttentionCNN, and DeepPPISP. This dataset provided us with an opportunity to extend our analysis beyond the confines of sequence-based datasets and directly compare the performance of these structure-based models against our own.

References

[1] S. Ansari and V. Helms. Statistical analysis of predominantly transient protein–protein interfaces. Proteins: Structure, Function, and Bioinformatics, 61(2):344–355, 2005.

[2] J. R. Bradford and D. R. Westhead. Improved prediction of protein–protein binding sites using a support vector machines approach. Bioinformatics, 21(8):1487–1494, 2005.

[3] X.-w. Chen and J. C. Jeong. Sequence-based prediction of protein interaction sites with an integrative method. Bioinformatics, 25(5):585–591, 2009.

[4] Y. Chen, J. Xu, B. Yang, Y. Zhao, and W. He. A novel method for prediction of protein interaction sites based on integrated rbf neural networks. Computers in biology and medicine, 42(4):402–407, 2012.

[5] A. Deng, H. Zhang, W. Wang, J. Zhang, D. Fan, P. Chen, and B. Wang. Developing computational model to predict protein-protein interaction sites based on the xgboost algorithm. International journal of molecular sciences, 21(7):2274, 2020.

[6] K. Dhole, G. Singh, P. P. Pai, and S. Mondal. Sequence-based prediction of protein-protein interaction sites with L1-logreg classifier. Journal of theoretical biology, 348:47–54, 2014.

[7] P. Gainza, F. Sverrisson, F. Monti, E. Rodola, D. Boscaini, M. Bronstein, and B. Correia. Deciphering interaction fingerprints from protein molecular surfaces using geometric deep learning. Nature Methods, 17(2):184–192, 2020.

[8] H. Guo, B. Liu, D. Cai, and T. Lu. Predicting protein–protein interaction sites using modified support vector machine. International Journal of Machine Learning and Cybernetics, 9:393–398, 2018.

[9] S. Hosseini and L. Ilie. PITHIA: Protein Interaction Site Prediction Using Multiple Sequence Alignments and Attention. International Journal of Molecular Sciences, 23(21):12814, 2022.

[10] J. Hu, M. Dong, Y.-X. Tang, and G.-J. Zhang. Improving protein-protein interaction site prediction using deep residual neural network. Analytical Biochemistry, 670:115132, 2023.

[11] Y. Kang, Y. Xu, X. Wang, B. Pu, X. Yang, Y. Rao, and J. Chen. Hn-ppisp: a hybrid network based on mlp-mixer for protein–protein interaction site prediction. Briefings in Bioinformatics, 24(1):bbac480, 2023.

[12] S. H. Khan, H. Tayara, and K. T. Chong. Prob-site: Protein binding site prediction using local features. Cells, 11(13):2117, 2022.

[13] K. Li, L. Quan, Y. Jiang, Y. Li, Y. Zhou, T. Wu, and Q. Lyu. ctp 2 isp: Protein–protein interaction sites prediction using convolution and transformer with data augmentation. IEEE/ACM Transactions on Computational Biology and Bioinformatics, 20(1):297–306, 2022.

[14] Y. Li, G. B. Golding, and L. Ilie. DELPHI: accurate deep ensemble model for protein interaction sites prediction. Bioinformatics, 37(7):896–904, 2021.

[15] Z. Li and J. Peng. Combining deep graph convolutional networks and prsa to enhance protein-protein interaction site prediction. In 2022 IEEE International Conference on Systems, Man, and Cybernetics (SMC), pages 2782–2789. IEEE, 2022.

[16] S. Lu, Y. Li, X. Nan, and S. Zhang. Attention-based convolutional neural networks for protein-protein interaction site prediction. In 2021 IEEE International Conference on Bioinformatics and Biomedicine (BIBM), pages 141–144. IEEE, 2021.

[17] S. Mahbub and M. S. Bayzid. Egret: edge aggregated graph attention networks and transfer learning improve protein–protein interaction site prediction. Briefings in Bioinformatics, 23(2):bbab578, 2022.

[18] M. Manfredi, C. Savojardo, P. L. Martelli, and R. Casadio. Ispred-seq: Deep neural networks and embeddings for predicting interaction sites in protein sequences. Journal of Molecular Biology, page 167963, 2023.

[19] Y. Murakami and K. Mizuguchi. Applying the Naïve Bayes classifier with kernel density estimation to the prediction of protein-protein interaction sites. Bioinformatics, 26(15):1841–1848, 2010.

[20] T. C. Northey, A. Barešić, and A. C. Martin. Intpred: a structure-based predictor of protein–protein interaction sites. Bioinformatics, 34(2):223–229, 2018.

[21] A. Porollo and J. Meller. Prediction-based fingerprints of protein–protein interactions. Proteins: Structure, Function, and Bioinformatics, 66(3):630–645, 2007.

[22] Z. Qiu and Q. Liu. Protein–protein interaction site prediction using random forest proximity distance. Journal of Bioinformatics and Computational Biology, 19(01):2050042, 2021.

[23] Z. Qiu, B. Zhou, and J. Yuan. Protein–protein interaction site predictions with minimum covariance determinant and mahalanobis distance. Journal of Theoretical Biology, 433:57–63, 2017.

[24] G. Singh, K. Dhole, P. P. Pai, and S. Mondal. Springs: prediction of protein-protein interaction sites using artificial neural networks. Technical report, PeerJ PrePrints, 2014.

[25] B. Stringer, H. de Ferrante, S. Abeln, J. Heringa, K. A. Feenstra, and R. Haydarlou. Pipenn: protein interface prediction from sequence with an ensemble of neural nets. Bioinformatics, 38(8):2111–2118, 2022.

[26] G. Taherzadeh, Y. Yang, T. Zhang, A. W.-C. Liew, and Y. Zhou. Sequence-based prediction of protein–peptide binding sites using support vector machine. Journal of computational chemistry, 37(13):1223–1229, 2016.

[27] M. Tang, L. Wu, X. Yu, Z. Chu, S. Jin, and J. Liu. Prediction of protein–protein interaction sites based on stratified attentional mechanisms. Frontiers in Genetics, 12:784863, 2021.

[28] M. A. Uddin and M. S. Ahmed. Modified naive bayes classifier for classification of protein-protein interaction sites. Journal of Bioscience and Agriculture Research, 26(02):2177–2184, 2020.

[29] T. Vreven, I. H. Moal, A. Vangone, B. G. Pierce, P. L. Kastritis, M. Torchala, R. Chaleil, B. Jiménez-García, P. A. Bates, J. Fernandez-Recio, et al. Updates to the integrated protein–protein interaction benchmarks: docking benchmark version 5 and affinity benchmark version 2. Journal of molecular biology, 427(19):3031–3041, 2015.

[30] P. Wang, G. Zhang, Z.-G. Yu, and G. Huang. A deep learning and xgboost-based method for predicting protein-protein interaction sites. Frontiers in Genetics, 12:752732, 2021.

[31] S. Wang, W. Chen, P. Han, X. Li, and T. Song. Rgn: Residue-based graph attention and convolutional network for protein–protein interaction site prediction. Journal of Chemical Information and Modeling, 62(23):5961–5974, 2022.

[32] X. Wang, Y. Zhang, B. Yu, A. Salhi, R. Chen, L. Wang, and Z. Liu. Prediction of protein-protein interaction sites through extreme gradient boosting with kernel principal component analysis. Computers in biology and medicine, 134:104516, 2021.

[33] Y. Wang, C. Mei, Y. Zhou, Y. Wang, C. Zheng, X. Zhen, Y. Xiong, P. Chen, J. Zhang, and B. Wang. Semi-supervised prediction of protein interaction sites from unlabeled sample information. BMC bioinformatics, 20(25):1–10, 2019.

[34] Z.-S. Wei, K. Han, J.-Y. Yang, H.-B. Shen, and D.-J. Yu. Protein–protein interaction sites prediction by ensembling svm and sample-weighted random forests. Neurocomputing, 193:201–212, 2016.

[35] Z.-S. Wei, J.-Y. Yang, H.-B. Shen, and D.-J. Yu. A cascade random forests algorithm for predicting protein-protein interaction sites. IEEE transactions on nanobioscience, 14(7):746–760, 2015.

[36] Z. Xie, X. Deng, and K. Shu. Prediction of protein–protein interaction sites using convolutional neural network and improved data sets. International journal of molecular sciences, 21(2):467, 2020.

[37] Q. Yuan, J. Chen, H. Zhao, Y. Zhou, and Y. Yang. Structure-aware protein–protein interaction site prediction using deep graph convolutional network. Bioinformatics, 38(1):125–132, 2022.

[38] M. Zeng, F. Zhang, F.-X. Wu, Y. Li, J. Wang, and M. Li. Protein–protein interaction site prediction through combining local and global features with deep neural networks. Bioinformatics, 36(4):1114–1120, 2020.

[39] B. Zhang, J. Li, L. Quan, Y. Chen, and Q. Lü. Sequence-based prediction of protein-protein interaction sites by simplified long short-term memory network. Neurocomputing, 357:86–100, 2019.

[40] J. Zhang and L. Kurgan. Scriber: accurate and partner type-specific prediction of protein-binding residues from proteins sequences. Bioinformatics, 35(14):i343–i353, 2019.

[41] H. Zhu, X. Du, and Y. Yao. Convsppis: identifying protein-protein interaction sites by an ensemble convolutional neural network with feature graph. Current Bioinformatics, 15(4):368–378, 2020.
